# Integrating Patient Choice and Collaborative Care Managers to Implement eHealth Tools in Depression: Self-Report Pilot Study

**DOI:** 10.2196/55349

**Published:** 2025-07-31

**Authors:** Jennifer Severe, Adrienne Lapidos, Danielle S Taubman, Amy Sochowicz, Sophia Wolk, Daniela Lopez, Abigail Biehl, Sagar V Parikh

**Affiliations:** 1 Department of Psychiatry, University of Michigan Medical School, 4250 Plymouth Road, Ann Arbor, MI, 48109, United States, 1 7347640231; 2 Eisenberg Family Depression Center, University of Michigan, Ann Arbor, MI, United States; 3 Institute for Healthcare Policy and Innovation, University of Michigan, Ann Arbor, MI, United States

**Keywords:** collaborative care model, e-mental health tools, primary care, depression, service delivery models, collaborative care, eHealth, tool, mental health, treatment, e-mental health, health technology, pilot study, self-help, self-care, self-management, self-report, behavioral therapy, female, women, clinic, evidence-based, principle, efficacy, attractiveness, low cost, mobile phone

## Abstract

**Background:**

Improving mental health treatment within the collaborative care model (CoCM) may be achieved by using e-mental health (e-MH) tools and addressing the challenges to their integration.

**Objective:**

This study aims to understand how patients select, engage, and use three self-help e-MH tools for depression, and to explore satisfaction with e-MH tools, with a particular emphasis on care manager interactions.

**Methods:**

This was a single-center, nonrandomized, preferred assignment study of two cognitive behavioral therapy–based tools (Moodkit and moodgym) and an educational website (the Depression Center Toolkit). The tools were recommended for use in 15-minute sessions 3 times a week, for 6 weeks, coupled with low-intensity care manager coaching. Utilization of e-MH was also captured during an additional 4 weeks without coaching. Self-report outcome measures were gathered at baseline, weekly for 6 weeks, at week 10, and through activities suggested by the tool.

**Results:**

The 32 participants enrolled were predominantly female (n=27, 84%), non-Hispanic Caucasian (n=29, 91%), with a mean age of 41.8 (SD 16.1; range 20 to 78) years. Most participants (n=26, 81%) presented with moderate to moderately severe depression (Patient Health Questionnaire-9=11‐19) and a marked level of impairment in different areas of functioning. About 81% (n=26) of the participants initially selected a cognitive behavioral therapy–based tool, and 19% (n=6) selected the educational website. In total, 4 of 32 (12%) participants switched tools within the first week, 6 of 32 (22%) participants dropped out, and one was removed. The remaining 25 active individuals used tools on average 3.0 (SD 2.4) times per week, most time (67%), for 11 to 20 minutes or more at a time. Of the 19 participants reached and surveyed at week 6, 52% (16/31) remained actively engaged with their tools, including 2 users who had switched tools and 8 between 45 and 78 years old. At week 10, about 75% (12/16) of this subgroup were using their tools with no coaching; this represented 49% of the cohort. Satisfaction increased with progressive use of the tool. The care manager’s low-intensity coaching lasted on average 7.9 (SD 3.9) minutes and promoted better understanding and greater use of the tools. Other facilitators to adherence consisted of organization, convenience, ease, accessibility, and privacy policies of the tools, while barriers included time constraints, depressive symptoms, and uncertainty about the efficacy of the tool.

**Conclusions:**

Uptake of e-MH tools for depression is feasible and associated with significant user satisfaction in CoCM. Low-intensity care manager coaching is consistent with the CoCM and is associated with uptake and ongoing use of e-MH tools. To our knowledge, this is the first study to leverage the care manager’s proactive outreach to and routine follow-ups with patients toward engagement in self-help digital tools.

## Introduction

### Background

**Figure 1.**
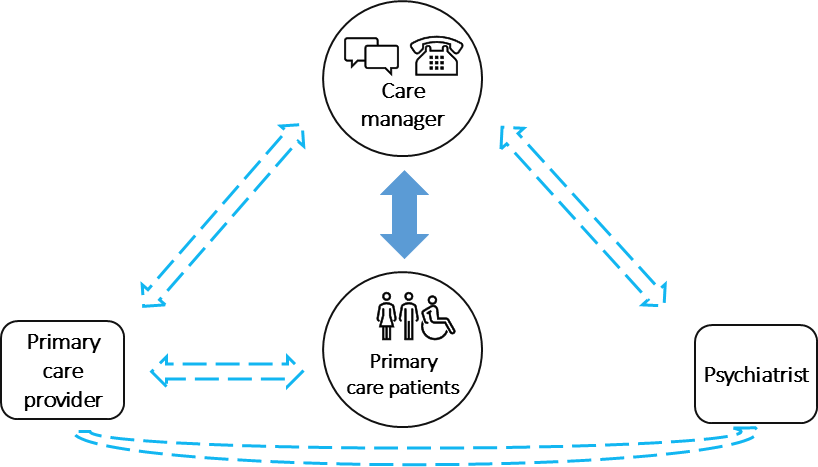
The collaborative care model.

Developed almost 3 decades ago, the collaborative care model (CoCM) is an extensively used evidence-based strategy for prompt access to quality behavioral health care in primary care settings [1-3]. Its positive impact on patient outcomes is supported by more than 80 randomized controlled trials [1] and relies largely on effective communication and teamwork between a primary care provider (PCP), a care manager, and a consulting psychiatrist (Figure 1). In this model, patients occupy a central place as empowered and active participants, making educated decisions about their treatment goals. The care manager, usually a trained social worker or nurse, maintains regular contact with patients through phone calls or secure SMS text messaging from enrollment in the CoCM to discharge. Through individualized care planning, the care manager routinely assesses psychosocial health needs, tracks patient outcomes through symptom measures, shares appropriate resources, discusses psychiatric recommendations, and provides support, as well as brief psychotherapy interventions.

To date, efforts to augment the CoCM with web-based and digital health technology have primarily focused on automated screening, measurement-based care, patient-provider communication, and, to a limited extent, basic psychoeducational information [4,5]. Integration of more active technology-augmented treatment, such as mental health therapy apps and websites, or “e-mental health (e-MH) tools,” is needed. These digital tools are self-help resources drawn from evidence-based psychotherapy principles [6] such as cognitive behavioral therapy (CBT) and from general coping strategies. They are accessible and affordable, and their effectiveness has been demonstrated through numerous randomized controlled trials, primarily for major depression [6,7]. While collaborative care clinicians show great enthusiasm and see value in adapting the CoCM for integration of e-MH tools, they also express doubts about clinic readiness [8] and point to similar challenges evidenced in digital health implementation studies, primarily poor user engagement [9-12], clinician’s lack of comfort, and perceived clinical burden [8-12].

This pilot study leveraged the dynamic relationship between patient and care manager within the CoCM to introduce three self-help e-MH tools for major depression into a primary care clinic: (1) moodgym, a web-based CBT program [13-15]; (2) MoodKit, a CBT smartphone app [16,17]; and (3) the Depression Center Toolkit, an educational website [18]. Given evidence of adherence challenges, mainly with standalone digital health interventions [5,9-11,19], these tools were added to the care manager’s therapeutic toolbox as self-help resources to discuss with patients during routine check-ins while adopting a low-intensity “coaching” style. We hypothesized this approach would enhance uptake and engagement in the e-MH tools, as Gordon et al [19] and Moon et al [5] suggested in their reviews of factors influencing real-world integration of health technology. Such an integration was most successful when operating within existing workflows. Considering the CoCM is designed such that it promotes patients’ abilities and confidence to manage their mental health, we encouraged patients to choose and switch their preferred tools based on their needs, a therapeutic method proven to scale up service uptake and engagement in clinical trials [20]. To our knowledge, this is the first study to optimize the unique patient-care manager dyad within the CoCM toward implementation of self-help e-MH tools for depression.

### Objectives

The aim of this pilot study was 2-fold. First, to assess user selection, uptake, and engagement style with three self-help e-MH tools. Second, to understand facilitators and barriers to user engagement with these tools, as well as user satisfaction, with and continued use of the intervention.

## Method

### Study Design

This was a single-center, nonrandomized, preference clinical trial of 3 self-help e-MH tools involving patients with major depression: moodgym, Moodkit, and the Depression Center Toolkit (Clinical Trial Registration number NCT04689568). The length of the intervention was 6 weeks, which was determined by the content of the tools and recommendations provided for their use. Our goal was to conduct the study in as naturalistic and patient-centered a manner as possible. As such, participants could choose the e-MH tools they preferred to try, with the option to switch tools if desired.

### Ethical Considerations

The University of Michigan Institutional Review Board approved and monitored the study (HUM00174081), and all procedures followed were in accordance with the ethical standards of the Institutional Review Board. The study complied with all regulations and policies regarding informed consent and the protection of personal information, privacy, and human rights. Participants had the ability to opt out of the study at any time.

### Study Setting and Participants

The study was conducted in a primary care clinic serving a catchment area with high needs for increased access and capacity for mental health services. The clinic CoCM program had the capacity to accept up to 100 patients on a rolling basis, managed by one full-time care manager (a licensed social worker), one psychiatrist, and multiple PCPs. The primary care clinic is part of an academic center.

Eligible participants were 18 years or older, English-speaking, newly referred to the clinic’s CoCM for major depression with a Patient Health Questionnaire-9 (PHQ-9) score of ≥11, and had access to the internet and a smartphone. Exclusion criteria consisted of already using an e-MH tool, cognitive impairment, substance use, and unstable mental or physical health problems (eg, marked suicidality and active psychosis).

### Study Recruitment

Participants were recruited between January 2021 and March 2022. The care manager performed a routine initial mental health evaluation for patients referred to the CoCM by their PCP. She used a script at the end of her assessment to ask pre-eligible patients whether the study coordinator could contact them about the study. A total of 5 months into recruitment, we streamlined the process by having the study coordinator directly contact the patients deemed pre-eligible based on their PHQ-9 scores or diagnosis of major depression at the time of their entry into the CoCM. We used an electronic consent form, which provided a detailed overview of the e-MH tools, including privacy policies.

To describe the sample profile at enrollment, we gathered information about demographics, mental health treatment, and comorbid conditions and assessed the level of impairment in various domains of functioning through the Sheehan Disability Scale. The PHQ-9 was also collected at week 6 as part of the care manager outcome monitoring to assess the sample’s clinical progress. Compensation was provided in the form of US $25 gift cards to participants after completing each of the enrollment, study completion (week 6), and postintervention (week 10) questionnaires.

### Study Interventions

The 3 self-help e-MH tools were selected based on their use of evidence-based principles, reported efficacy, attractiveness of user interface, and low cost [13-18]. Participants received a descriptive summary of the study interventions, including general recommendations on daily or weekly use (Multimedia Appendix 1).

moodgym is an interactive CBT-based program, accessible on any electronic device, designed to prevent or reduce symptoms of depression and anxiety, and encourage good coping skills [13-15]. moodgym consists of 5 modules that are completed in a prescribed order and through animated demonstrations, mood tracking, homework exercises, and feedback that helps users identify and manage different thinking styles and vulnerabilities and their impact on emotions and outcomes. The study covered the annual subscription cost for moodgym (US $27). The study recommendation for use stated, “Aim to complete one module on this program weekly.”

MoodKit is a CBT-based smartphone app, available only through the Apple Store, that provides integrated tools to effectively manage symptoms of depression and anxiety, enhance emotional well-being, and more significantly, coping self-efficacy [16,17]. MoodKit offers four features in an unstructured, unprompted way, comprising a collection of mood-enhancing activities and suggested steps for accomplishing them, a thought checker, a mood tracker, and a variety of therapeutically designed journal templates. The study covered the one-time fee for MoodKit (US $4.99). The study recommendation for use stated, “Aim to complete one individual goal and use two sections of this app once daily.”

The Depression Center Toolkit is an informational website developed by academic experts with the help of individuals with lived experience [18]. The Toolkit is divided into 5 sections that focus on different aspects of someone’s mental health journey. It includes detailed educational information, strategies to incorporate into a health regimen, steps toward making lasting lifestyle changes, and practical tools such as communication guides, medication tracking journals, and sleep logs. The Toolkit is free and accessible on any device. The study recommendation for use stated, “Focus on one section a week, switch each week*.*”

The care manager incorporated a guided script of low-intensity coaching into her weekly routine check-ins with participants. It was designed to remediate likely mechanisms underlying nonadherence to the e-MH tools [8-11], which included resolving minor technical questions, supplementing information and suggestions with emotional support, and motivating continued use of the tools (Multimedia Appendix 1).

### Study Outcomes

#### Primary Outcomes

Based on the content of the self-help tools and instructions provided for their use, we conceptualized engagement as the use of the tool three times a week, for approximately 15 minutes at a time. Once a week, for 6 weeks, the care manager gathered frequency and duration of use of the tools through participants’ self-reports.

#### Secondary Outcomes

Facilitators and barriers to user engagement, as well as user satisfaction with the tools, were collected once weekly, for 6 weeks, through participants’ self-reports as part of the care manager’s scripted low-intensity coaching, which included open-ended questions for discussion (Multimedia Appendix 1). We reviewed all write-in qualitative responses as a team, and using discussion and consensus, identified salient themes. After the study completion at week 10, the care manager surveyed participants on their continued use of their tools.

### Data Analysis

The care manager gathered the study outcomes during her routine check-ins with patients. In controlled and real-world settings, the care manager’s inability to reach patients for a routine check-in is common and can be as high as 36% [21]. We reviewed the trend of missed check-ins at the study site, and using discussion and consensus as a team, agreed that missingness did not differ during and after the pilot study. The care manager usually explores reasons around missed check-ins as part of the CoCM personalized care plan to ensure patient time is respected. For this real-world pilot, participants were kept in the study regardless of the number of check-ins missed unless they asked to be removed from the study. We deemed it useful not to impute the missing data and kept our observation of the study events unchanged. As a self-report study, there was a risk of social desirability bias, which we tried to minimize through the care manager’s weekly monitoring and discussion of patients’ progress with homework or activities suggested by their tools or the care manager. We used descriptive statistics to report individual survey responses and their relative percentages. We also provided narrative summaries of participant comments.

## Results

### Study Recruitment

A total of 121 patients were found pre-eligible for the study, and 115 patients qualified. Among those, 51 patients were unreachable, 32 patients consented to participate in the study, and 32 patients declined and were encouraged to share why they were “not interested” or asked to be recontacted later. About 37% (12/32) had issues with the study design including the lack of focus on anxiety disorders, the lack of diversity of apps (such as meditation-based app), and the length of the consent process; 28% (9/32) were busy with either caregiving duties or work or managing an acute illness; 9% (3/32) pointed to their lack of digital skills; and 25% (8/32) provided no explanation. An additional 495 individuals who saw the study posted on the academic center’s health research website expressed interest in participating, but none qualified as they were not part of the study’s primary clinic.

### Study Participants

The 32 enrolled participants were primarily identified as female (27/32, 84%) and White, non-Hispanic (31/32, 97%), which reflected the racial and ethnic background of the study catchment area. The mean age was 41.8 (SD 16.1; range: 20-78) years. About 60% (19/32) of the sample were in a relationship, 50% (16/32) had children, and 66% (21/32) were employed. The mean PHQ-9 score was 16 (SD 3.7; range: 11‐23) at the time of enrollment. Based on the participants’ clinical presentation, the majority needed psychotherapy (29/32, 91%), and all needed pharmacotherapy interventions. About 88% (28/32) reported comorbid anxiety symptoms, and 66% (21/32) endorsed active pain (Table 1).

**Table 1.** Characteristics of the study sample (n=32).

Initial e-MH^a^ tool selection		Moodkit (n=17)	moodgym (n=8)	Depression toolkit (n=7)	Sample total (n=32)
Sex, n (%)					
Female		11 (34)	9 (28)	7 (22)	27 (84)
Male		3 (9)	2 (6)	0 (0)	5 (16)
Age (years)					
Mean (SD)		44.6 (18.3)	38 (11.6)	44.2 (15.8)	41.8 (16.1)
Range		20‐78	24‐62	20‐60	20‐78
Race and ethnicity, n (%)					
Asian		0 (0)	0 (0)	1 (3)	1 (3)
White, non-Hispanic		17 (53)	8 (25)	6 (19)	31 (97)
Marital status, n (%)					
In a relationship		8 (25)	7 (22)	4 (13)	19 (60)
Not in a relationship		6 (19)	4 (12)	3 (9)	10 (40)
Caring for young children, n (%)					
Yes		9 (28)	5 (15)	2 (6)	16 (50)
No		5 (16)	6 (19)	5 (16)	16 (50)
Employment status, n (%)					
Employed full-time or part-time		8 (25)	8 (25)	5 (16)	21 (66)
Unemployed		3 (9)	3 (9)	2 (7)	8 (25)
Retired		3 (9)	0 (0)	0 (0)	3 (9)
PHQ-9^b^ severity					
Mean (SD)		16.5 (4.0)	14.8 (3.1)	16.6 (3.6)	16 (3.7)
Range		11‐23	11‐21	12‐22	11‐23
Need a referral to psychotherapy, n (%)					
Yes		13 (41)	10 (31)	6 (19)	29 (91)
No		1 (3)	1 (3)	1 (3)	3 (9)
Need antidepressant adjustment or initiation, n (%)					
Yes		13 (41)	10 (31)	7 (22)	30 (94)
No		1 (3)	1 (3)	0 (5)	2 (6)

a e-MH: e-mental health.

b PHQ-9: Patient Health Questionnaire-9.

A total of 29 participants filled out the Sheehan Disability Scale and reported a “markedly impaired to extremely impaired functioning” in social life (17/29, 59%), in family life and home responsibilities (16/29, 55%), and at work and in school activities (10/29, 35%).

### Study Interventions

The CBT app, MoodKit, was initially selected by 53% (n=17) of the participants, the CBT web-based program, moodgym, by 25% (n=8) of participants, and the educational website, the Depression Center Toolkit, by 22% (n=7) of participants (Table 1).

In total, 4 (12%) participants requested to switch tools within the first week, including 3 users who could not access MoodKit through their Android operating system and opted to transition to moodgym (n=2) and the Depression Center Toolkit (n=1). Another participant, interested in a “more interactive” tool “with journaling” option, changed from the Depression Center Toolkit to moodgym.

The care manager made 162 calls to participants as part of her routine clinical check-ins and weekly low-intensity coaching of the tools, at which time she also gathered some of the study outcomes. Of these 162 calls, 77% (125/162) were successful, 20% (33/162) were marked “missed” for participants who were unreachable on a given week for various reasons including illnesses and caregiving responsibilities, and 3% (4/162) were solely made to discharge participants from the study at week 1 (Table 2).

**Table 2.** Frequency of use of participants’ selected e-MH tools.

Final e-MH^a^ tool selection		Moodkit (n=13^b^)	moodgym (n=11)	Depression toolkit (n=7)	Sample total (n=31^b^)
Participants who withdrew from the study, n (%)		4 (13)	2 (6)	0 (0)	6 (19)
Use of the tools					
Week 1					
Mean (SD)		4.2 (4.3)	2.7 (1.5)	2.0 (1.4)	3.2 (3.2)
Range		0‐15	1‐5	0‐4	0‐15
Week 2					
Mean (SD)		3.8 (2.2)	2.6 (2.0)	2.2 (1.7)	3.0 (2.1)
Range		0‐7	1‐7	0‐4	0‐7
Week 3	
Mean (SD)		3.5 (2.6)	1.8 (0.4)	3.6 (2.1)	3.1 (2.2)
Range		0‐8	1‐2	1‐7	0‐8
Week 4					
Mean (SD)		5.0 (2.1)	2.6 (1.8)	1.4 (1.5)	3.2 (2.4)
Range		2‐7	0‐6	0‐4	0‐7
Week 5					
Mean (SD)		3.6 (2.2)	1.8 (0.4)	3.3 (1.9)	3.1 (2.0)
Range		0‐7	1‐2	1‐7	0‐7
Week 6					
Mean (SD)		2.7 (2.1)	1.6 (0.8)	3.4 (2.1)	2.6 (1.9)
Range		0‐7	0‐2	1‐7	0‐7
Total					
Mean (SD)		3.8 (2.9)	2.3 (1.5)	2.7 (2.0)	3.0 (2.4)
Range		0‐15	0‐7	0‐7	0‐15
Participants reached, n (%)					
Week 1		11 (85)	7 (64)	6 (86)	24 (77)
Week 2		9 (70)	8 (73)	6 (86)	23 (74)
Week 3		11 (85)	6 (55)	5 (71)	22 (65)
Week 4		7 (54)	7 (64)	5 (71)	19 (61)
Week 5		8 (62)	4 (36)	6 (86)	18 (58)
Week 6		9 (70)	5 (45)	5 (71)	19 (61)

a e-MH: e-mental health.

b One participant was removed by the study team at week 1, which brought the new sample size to 31 (n=31).

The low-intensity coaching lasted on average 7.9 (SD 3.9; range: 2‐20) minutes, and 90% (112/125) of the calls varied between 2 and 10 minutes; 9% (11/125) ran for 15 minutes for participants with engagement issues, and 1% (2/162) lasted 20 minutes for one participant who wanted to better understand the concept of cognitive restructuring proposed by the tool. The average time for the brief coaching varies minimally per week whether it was the care manager’s first (8.1 min), second (8.1 min), third (7.9 min), fourth (7.8 min), fifth (7.9 min), or sixth (7.8 min) calls to participants to discuss engagement with their selected tools.

### Primary Outcomes

A total of 6 of 32 (19%) participants withdrew from the study (Table 1) and did so within the first week (n=3), second week (n=1), and third week (n=2). They were between 30 and 76 years old (n=6), had initially selected MoodKit (n=4) or moodgym (n=2), did not ask to switch tools (n=6), and pointed primarily to time constraints with family life, home responsibilities, or work as the main reasons for dropout (n=4). One participant deemed not appropriate for the study was discharged at week 1, which brought the sample size to 31.

The care manager reached between 58% (18/31) and 77% (24/31) of the study sample weekly, who reported interacting with their tools on average between 3.0 and 3.2 times the first 5 weeks, and 2.6 times during the last week of monitoring, week 6 (Table 2). Weekly engagement with the tools varied between none to seven times weekly, with one Moodkit user reporting engaging with the app 15 times the first week and 8 times the third week for a more in-depth exploration and practice of the CBT activities. Average utilization was higher for Moodkit, followed by the Depression Center Toolkit, then moodgym (Table 2).

For the most part, participants interacted with their tools “11 to 20 minutes” at a time (52/125, 42%) and “more than 20 minutes” at a time (31/125, 25%). The tools were also used for “6 to 10 minutes” about 19% (24/125) of the time, “1 to 5 minutes” about 3% (4/125) of the time, and none about 11% (14/125) of the time. More time (“11 min or greater”) was spent interacting with Moodkit, compared to moodgym and the Depression Center Toolkit (Table 3).

**Table 3.** Duration of use of participants’ selected e-MH^a^ tools.

Final e-MH tool selection	Moodkit (n=13)^b^	moodgym (n=11)	Depression toolkit (n=7)	Sample total (n=31)^b^
Number of times participants were reached, n	55	37	33	125
Number of times participants use the tool for a designated time frame (minutes), n (%)				
0	7 (5.6)	2 (1.6)	5 (4)	14 (11.2)
1-5	3 (2.4)	1 (0.8)	0 (0)	4 (3.2)
6-10	12 (9.6)	4 (3.2)	8 (6.4)	24 (19.2)
11-20	24 (19.2)	14 (11.2)	14 (11.2)	52 (41.6)
More than 20	9 (7.2)	16 (12.8)	6 (4.8)	31 (24.8)

a e-MH: e-mental health.

b One participant was removed by the study team at week 1 which brought the new sample size to 31 (n=31).

### Continuous Engagement at Study Completion and Postintervention

A total of 19 of 31 (61%) participants were reached and surveyed at the end of the study, at week 6. Of these, 16 (52%) participants were still using their tools on average 3.1 (SD 1.8; range: 1‐7) times for at least 11 minutes at a time for the most part, including 2 users who had switched tools. Compared to the rest of the study sample, they missed fewer check-ins with the care manager (6/96, 6%), reported greater interactions on average with their tools (3.6 times) over a longer period of time (5 or 6 wk), and endorsed a lower depression score at study completion (6.8, SD 5.0; range: 1‐15) compared to baseline (15.5, SD 3.6; range: 11‐21). Half (8/16) of these users were between 45 and 78 years old. Four weeks postintervention, at week 10, a total of 12 participants in this subgroup reported continued use of the interventions with no coaching; this represented 39% (12/31) of the study sample.

About half of the study sample (17/31, 55%) completed the PHQ-9 at the end of the study. Their average depression severity score decreased to 8.1 (SD 5.7; range: 1‐22) compared to 16 (SD 3.7; range: 11‐23) at enrollment.

### Secondary Outcomes

Participants’ satisfaction with their selected tools increased with time. Satisfaction with the selected e-MH tools was reported for users (21/31, 68%) who did a moderate amount to a great deal of homework or activities suggested by their tools or the care manager. Dissatisfaction or uncertainty with the e-MH tools was reported for users who did not engage or interacted very little with their tools and put a great focus on difficulties navigating the tool or time constraints.

Participant-reported facilitators consisted of some specific characteristics of each tool, such as organization, convenience, accessibility, and privacy policies. Participant-reported barriers to adherence to the tools included time constraints, depressive symptoms, and uncertainty about the efficacy of the tool.

### Participant Engagement With the CoCM

A total of 8 of 31 (25%) study participants minimally engaged with the CoCM during and after the study. They missed the care manager’s calls or asked to be recontacted on numerous occasions. They were discharged by the collaborative care team within 3 to 6 months of enrollment in the CoCM. In total, 4 of these 8 patients had requested to be removed from the study within the first 2 weeks, and the other 4 patients remained unreachable for most of the study.

## Discussion

### Principal Findings

This was a real-world pilot study based in a CoCM primary care program, assessing patients’ selection, uptake, and engagement with 2 CBT-based digital tools, Moodkit and moodgym, and an educational website, the Depression Center Toolkit. The study interventions were well-received, with a preference given to the CBT-based tools. All 3 tools were paired with adherence-focused, low-intensity coaching incorporated into the care manager’s routine check-ins with patients. Our study sample consisted primarily of non-Hispanic White, partnered women with childcare or work responsibilities who endorsed moderate to severe major depression (average PHQ-9 score: 16, SD 3.7), comorbid anxiety and pain symptoms, and needed psychotherapy referrals. Participants identified parental duties and acute illnesses as the chief reasons for missing 20% of the care manager’s weekly follow-ups and study outcome tracking. However, designed to be a real-world patient-centered intervention, participants were allowed to continue in the study as they would in the CoCM, where missingness is not uncommon [21], and engagement is known to be particularly challenging for caregiver mothers with mental illnesses [22].

The care manager reached 58% (18/31) to 77% (24/31) of the study sample weekly. Participants reported engaging with the interventions on average 3 to 3.2 times per week for the first 5 weeks, and 2.6 times during the last week. Most (67%) did so for 11 minutes or more at a time. Such a decline in frequency of use is expected and has been observed in numerous digital mental health studies [10,11,14,15,19,23-26], including those involving moodgym [[[[[[[[[[14,15]]]]]]]]]] and Moodkit [25]. We selected our engagement target with this in mind and designed the low-intensity coaching to encourage what Zhang et al [24] called the right dose of digital therapy (“not too much or not too little”) while promoting three key user behaviors essential to clinically meaningful use of e-MH tools: learning (eg, identifying a coping activity), goal setting, and self-tracking (eg, mood rate and sleep logs). While this pilot did not intend to assess the clinical efficacy of the tools, participants’ depression severity scores improved at the end of the study. Such an improvement was greater (16/31, 52%) for those with consistent use of tools until the end of the study, at week 6. This continuous engagement style was comparable to [14,25] or higher than [14,15,26,24] previous digital mental health interventions with moodgym, Moodkit, and self-report pilot trials, some conducted in primary care clinics. Retention rates at 5 and 6 weeks were as low as 26.5% even when these interventions were coupled with a digital health support. Pharmacotherapy management, being an integral part of the CoCM, cannot be ruled out as a contributor to the PHQ-9 score improvement.

### Shared Familiarity With the e-MH Tools

The study team’s knowledge of available e-MH tools was essential to the intervention selection process. A shared familiarity with the tools facilitated the development of a reasonable and therapeutic engagement target, as well as suggestions for maximization of their use. It also promoted informed decision-making. Patients received a descriptive summary of the tools with recommendations on how to use them before selecting one. Tools with evidence-based principles, reported efficacy, an attractive user interface, low cost, and no embedded clinician support were given priority for inclusion in the study. Overall, participants’ satisfaction with the tools increased with time and was observed for those who engaged in homework or activities suggested by their tools or the care manager, who became accustomed to the different modules.

### Existing Team-Based Care and Workflows

Capitalizing on the existing infrastructure of the CoCM for integration of the tools allowed for an adjunct care approach with monitoring and follow-ups and saved the cost, time, and clinical burden that may have ensued with a standalone implementation process. For the study duration, the CoCM workflow was adjusted as minimally as possible. As such, the care manager carried out her usual clinical duties in coordinating care with participants’ PCPs and their consulting psychiatrist and also shared patients’ experiences with their selected tools. The low-intensity “coaching” lasted on average 7.8 to 8.1 minutes, regardless of the study week. Our overall observation is that the care manager perceived no additional burden with the intervention. The latter seemed to have effectively supplemented her brief psychotherapy interventions by offering a collection of activities and tools to which patients might otherwise not have had access, such as mood tracking, journaling, homework exercises, advice for lifestyle changes, medication, and sleep logs.

### Flexible and Tailored Approach to Engagement

In contrast to other digital health implementation studies, patients were encouraged to select their preferred tools and switch if desired. In total, 4 participants took advantage of this flexible approach because of phone compatibility issues or a lack of therapeutic functionality with the selected tools. This approach is consistent with centering patient empowerment and shared decision-making in the CoCM and is also more naturalistic, given that in real-life settings, it is unlikely that clinics would require patients to choose particular tools. A potential key determinant to uptake and engagement to consider is the appropriate timing for the introduction of e-MH tools into patient care. Patients with momentary time constraints, due to an acute illness or a specific life event, who did not enroll were asked to be recontacted to enroll, while those already in the study stopped using their tools for 1 or 2 weeks. We accommodated both. Timing should be explored in concert with the patient.

As part of the personalized care planning offered in the CoCM, there is value in inquiring about why patients declined self-help digital health resources. For instance, most patients (12/32, 37%) who turned down participation in the study pointed to a lack of focus on comorbid anxiety disorders and the absence of meditation-based apps, among other issues. Relatedly, about 88% (22/32) of the study sample were found with active anxiety symptoms of various severity in addition to major depression. A more diverse menu of e-MH tools could be beneficial in the future, including apps for pain, another active comorbidity found in 66% (21/32) of the study sample.

### Limitations

Due to the small sample size, we were not able to make statistical inferences. In total, 20% of the care manager’s check-ins were missed as patients were unreachable for various reasons, resulting in a missed opportunity to deliver the low-intensity coaching and gather the study outcomes. With the self-report design of this pilot study, we faced the possibility of social desirability bias, which we tried to minimize by weekly monitoring and discussion of patients’ progress with homework or activities suggested by their tools or the care manager.

### Conclusions

Efforts to augment the CoCM with web-based and digital health technology have been limited. To our knowledge, this is the first study to leverage the unique patient-care manager relationship within the CoCM toward integration of self-help e-MH tools for depression. Such an implementation can be successful when centered around patient empowerment and integrated within existing clinical workflows. These findings may inform digital health intervention efforts in the CoCM with considerations for the barriers that are unique to this model.

## Supplementary material

10.2196/55349 Multimedia Appendix 1 Study Outcome Questionnaire.

## Abbreviations

CBT: cognitive behavioral therapy
CoCM: collaborative care model
e-MH: e-mental health
PCP: primary care provider
PHQ-9: Patient Health Questionnaire-9
