# Supplementary material for: Integrating Patient Choice and Collaborative Care Managers to Implement eHealth Tools in Depression: Self-Report Pilot Study
Source: J Particip Med. 2025 Jul 31;17:e55349. doi: 10.2196/55349 (PMC12313309; doi:10.2196/55349)
Supplement: Multimedia Appendix 1 [file jopm-v17-e55349-s001.pdf]

Support call checklist

Participant ID number

____

Week #

○ 1

○ 2

○ 3

○ 4

○ 5

○ 6

Date of call

____

Outcome of call

○ Participant was reached

○ Participant was not reached

Display This Question:

If Outcome of call = Participant was reached

Call type

○ First call

○ Follow-up call

End of Block: Intro block

Start of Block: Follow up call block

Display This Question:

If Call type = First call

Which self-help tool did you sign up for?

◯ Moodkit

◯ Moodgym

◯ Depression Center Toolkit

Display This Question:

If Call type = First call

What made you decide to choose that one over the others? (Enter brief notes based on response)

____

Did you use the tool in the past week?

◯ Yes

◯ No

Display This Question:

If Did you use the tool in the past week? = Yes

About how many times did you use it?

____

Display This Question:

If Did you use the tool in the past week? = Yes

Typically, how long did you spend using it each time? [Note: make sure the person understands we are asking the person how long they spent on average each time, not how long they spent over the whole week]

- ◯ 1-5 minutes
- ◯ 6-10 minutes
- ◯ 11-20 minutes
- ◯ More than 20 minutes

Display This Question:

If Did you use the tool in the past week? = Yes

Did you do any homework or extra activities suggested by your tool, like writing down your thoughts or doing activities you enjoy?

- ◯ A great deal
- ◯ A lot
- ◯ A moderate amount
- ◯ A little
- ◯ None at all

Display This Question:

If Did you use the tool in the past week? = Yes

How satisfied have you been with the tool so far? Would you say you've been...

- ◯ Extremely satisfied
- ◯ Somewhat satisfied
- ◯ Neither satisfied nor dissatisfied
- ◯ Somewhat dissatisfied
- ◯ Extremely dissatisfied

Display This Question:

If Did you use the tool in the past week? = Yes

Tell me a little about what makes you feel satisfied or unsatisfied with the tool.

____

Display This Question:

If How satisfied have you been with the tool so far? Would you say you've been... = Extremely satisfied

Or How satisfied have you been with the tool so far? Would you say you've been... = Somewhat satisfied

Provide encouragement and motivational support for the person to continue to use the tool, and then end the call. Discuss barriers and facilitators. Affirm and reflect progress.

Display This Question:

If How satisfied have you been with the tool so far? Would you say you've been... = Neither satisfied nor dissatisfied

Or How satisfied have you been with the tool so far? Would you say you've been... = Somewhat dissatisfied

Or How satisfied have you been with the tool so far? Would you say you've been... = Extremely dissatisfied

Tell me a bit about what is not working well with the tool. (Select all choices that match participant's natural response)

- ☐ Not interested
- ☐ Not enough time
- ☐ Did not think it would help
- ☐ Technical problem
- ☐ Depression sxs (e.g. amotivation, fatigue, concentration, etc.)
- ☐ Other ____

Display This Question:

If How satisfied have you been with the tool so far? Would you say you've been... = Neither satisfied nor dissatisfied

Or How satisfied have you been with the tool so far? Would you say you've been... = Somewhat dissatisfied

Or How satisfied have you been with the tool so far? Would you say you've been... = Extremely dissatisfied

Briefly validate stated barriers, then provide concrete and/or motivational support as needed. Affirm and reflect progress. Below please select the type of support you provided. Then thank the person and end the call.

- ☐ Tried to spark interest in the tool
- ☐ Trouble shooting timing issues, like choosing time of day
- ☐ Provided information about evidence that the tool may help
- ☐ Technical support
- ☐ Other ____

Display This Question:

If Did you use the tool in the past week? = No

Tell me a bit about what gets in the way of using it. (Select all choices that match participant's natural response)

- [ ] Not interested
- [ ] Not enough time
- [ ] Did not think it would help
- [ ] Tried it before and did not like it
- [ ] Technical problem
- [ ] Depression sxs (e.g. amotivation, fatigue, concentration, etc.)
- [ ] Other ____

Display This Question:

If Did you use the tool in the past week? = No

Briefly validate stated barriers, then provide concrete and/or motivational support as needed. Affirm and reflect progress. Below please select the type of support you provided. Then thank the person and end the call.

- [ ] Tried to spark interest in the tool
- [ ] Trouble shooting timing issues, like choosing a time of day
- [ ] Provided information about evidence that the tool may help
- [ ] Technical support
- [ ] Other ____

Length of call in minutes
